# Tetravalent antibodies are more potent and efficacious erythropoiesis‐stimulating agents than erythropoietin in vivo

**DOI:** 10.1002/pro.70462

**Published:** 2026-01-20

**Authors:** Jarrett J. Adams, Levi L. Blazer, Jacky Chung, Minoo Karimi, Taylor Davidson, Bailey Blair, Carlos Waddle, Craig A. Hokanson, Heather A. Bruce, Alexander U. Singer, Eva‐Maria Tombak, Kiira Gildemann, Nele Tamberg, Kaja Kiiver, Mart Ustav, Yue Ma, Luigi Colombo, Lily Jun‐Shen Huang, Stephen W. Michnick, Orson W. Moe, Sachdev S. Sidhu

**Affiliations:** ^1^ Anvil Institute for Systems Biologics Toronto Ontario Canada; ^2^ EPOK Therapeutics Toronto Ontario Canada; ^3^ Simisco Biosciences Hayward California USA; ^4^ Département de Biochimie Université de Montréal Montréal Québec Canada; ^5^ Departments of Internal Medicine and Physiology University of Texas Southwestern Medical Center Dallas Texas USA; ^6^ Icosagen Tartu Estonia

**Keywords:** agonist, antibody, antibody polymer, diabody, dimer, drug design, EPO, EPOR, erythropoiesis, helical ultrastructure, protein engineering

## Abstract

Recent studies have shown that tetravalent antibodies are potent and efficacious agonists of the erythropoietin (EPO) receptor (EPOR) both in vitro and in vivo. To identify antibody‐based erythropoiesis‐stimulating agents (ESAs) with therapeutic potential, we evaluated various tetravalent antibody formats for EPOR agonism and key biophysical properties necessary for biologic drug development. We identified two distinct tetravalent antibody formats that strongly stimulated the growth of UT7/Epo cells, which rely on EPOR signaling for proliferation. Moreover, one of these formats exhibited ideal biophysical characteristics for drug development. This format consisted of a diabody (Db) and two antigen‐binding fragment (Fab) arms fused to the N‐ and C‐termini of an Fc domain, respectively, to form a tetravalent Db‐Fc‐Fab (EPRA‐0322). In a mouse model expressing the human EPOR, EPRA‐0322 induced erythropoiesis with greater potency, efficacy, and duration than darbepoetin, a hyperglycosylated EPO currently used in clinical practice. These findings highlight tetravalent antibodies, and the Db‐Fc‐Fab format in particular, as promising next‐generation ESAs suitable for large‐scale production and clinical use.

## 1 INTRODUCTION

The cytokine erythropoietin (EPO) promotes red blood cell production by activating the erythropoietin receptor (EPOR) through dimerization (Chida et al., 1999; Lykov, 2024; Zhang et al., 2014). Recombinant EPO and its derivatives have been widely used for decades as erythropoiesis‐stimulating agents (ESAs) to treat anemia, particularly in patients with chronic kidney disease (CKD) (Bunn, 2007; Chida et al., 1999; Chung et al., 2023). However, EPO‐based therapies have several limitations, including high manufacturing costs, poor thermal stability, and short in vivo half‐lives (Doshi et al., 2013; Hodson & Strippoli, 2021). Moreover, EPO has been proposed to interact with receptors beyond EPOR, such as the *β*‐common light chain receptor (*β*c/CD131) (Bohr et al., 2015) and the receptor tyrosine kinase EPHB4 (Pradeep et al., 2015). These additional interactions may contribute to undesirable effects, including tumorigenic and cardiovascular risks (Bennett et al., 2016; Peng et al., 2020). Therefore, there is a critical need to develop alternative ESAs that match or surpass the efficacy of EPO while minimizing its drawbacks.

Monoclonal antibodies (Abs) are attractive therapeutic agents due to their favorable properties, including high stability, scalable production, long circulating half‐lives, and low immunogenicity (Crescioli et al., 2025). However, despite substantial efforts (Liu et al., 2007; Zhang et al., 2012), no Ab‐based ESA has yet reached clinical testing. We recently demonstrated that the conventional bivalent IgG format — and other bivalent Ab configurations — is inherently limited in its ability to activate EPOR, primarily due to steric constraints that prevent the precise receptor dimerization required for signaling (Adams et al., 2025). In contrast, we showed that a tetravalent Ab, or tetrabody (Tb) — which was present as a minor component in bivalent diabody (Db) preparations — was a highly active agonist of human EPOR both in vitro and in vivo (Adams et al., 2025). Structural modeling suggested that the Tb could arrange EPORs in an asymmetric dimer configuration closely resembling that induced by EPO, while the Db alone could not.

Although this discovery opened new avenues for developing Ab‐based ESAs, the Tb format presented challenges related to production and stability. It was difficult to manufacture at scale and preparations exhibited heterogeneity due to interconversion between oligomeric states — issues that made it incompatible with clinical‐grade drug development.

In this study, we built on these findings and took further steps toward ESA drug development by engineering tetravalent Ab formats that can be stably and homogenously produced, as opposed to spontaneously forming as a minor component within a bivalent preparation. We incorporated our previously validated EPOR‐binding sequences into these constructs and identified several formats that could be produced at high yield and purity. Two of these formats showed high potency and efficacy in vitro and outperformed EPO in vivo. Notably, we demonstrated that one of these formats — the Db‐Fc‐Fab, which has already been used to develop a clinical‐stage biologic currently undergoing phase 2b/3 trials (NCT06571045) — was a potent tetravalent EPOR agonist (EPRA‐0322) that could be produced as a stable, homogenous preparation. Our present study therefore resolves issues described in our previous paper and paves the way for clinical development of a novel ESA with improved efficiency and lower side effects than currently available anemia treatments.

## 2 RESULTS

### 2.1 Effects of *V*_L_ – *V*_H_ oligomerization on EPOR agonist activity

We previously used a synthetic phage‐displayed Db library to select anti‐EPOR Dbs and identified a clone (variant 1) that was used to engineer derivatives with changes restricted to the complementarity‐determining regions (CDRs) (Figure S1A) (Adams et al., 2025). These derivatives varied in their affinities and propensities to form higher order oligomers. For this study, we chose variant 1.4 as a modular scaffold to construct tetravalent Abs.

**FIGURE 1:**
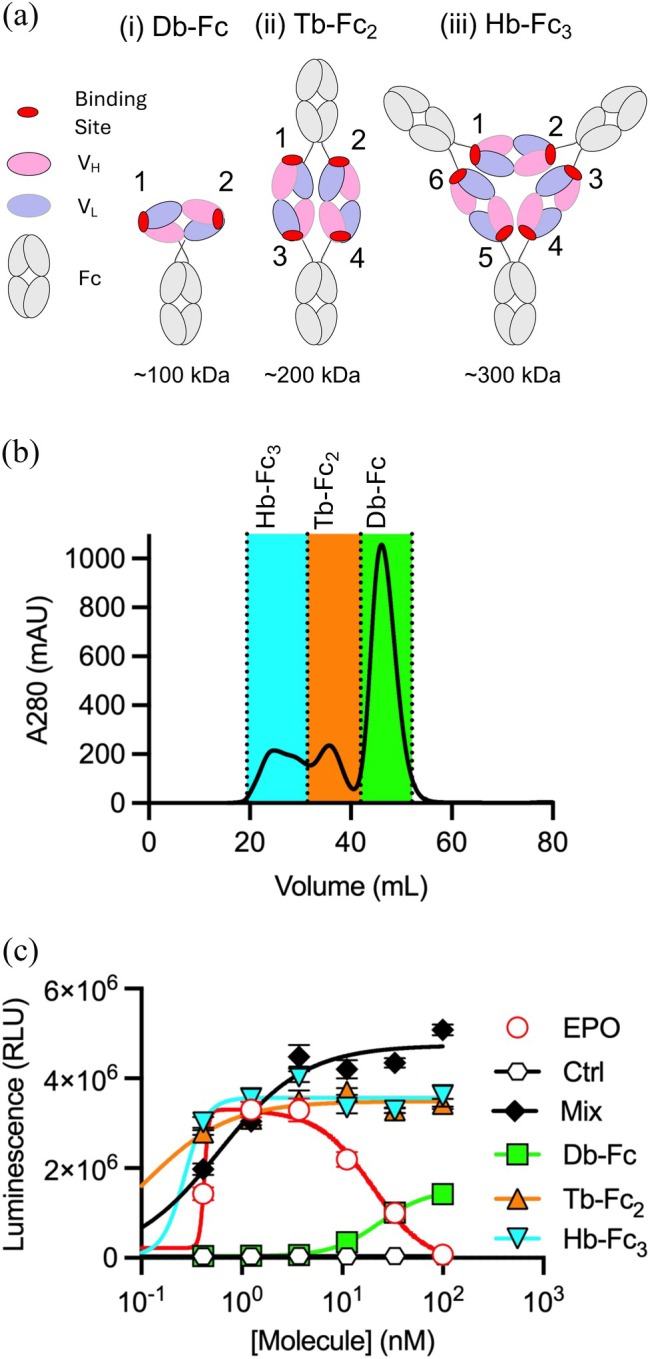
Effects of *V*_L_–*V*_H_ 1.4 oligomerization on EPOR agonist activity. (A) The following Ab formats are shown: (i) bivalent Db‐Fc, (ii) tetravalent Tb‐Fc_2_, and (iii) hexavalent Hb‐Fc_3_. The following colors are used: CDRs (red), *V*_H_ (pink), *V*_L_ (lavender), and Fc (gray). (B) SEC of *V*_L_–*V*_H_ 1.4 fused to an Fc domain on a preparative Superdex 200 column. Protein peaks were collected as 3 fractions indicated by dotted lines representing putative Db‐Fc (green), Tb‐Fc_2_ (orange), or Hb‐Fc_3_ (cyan). (C) UT‐7/Epo cell proliferation assays. Proliferation (*y*‐axis) was monitored in the presence of various concentrations (*x*‐axis) of positive control EPO (unfilled red circles), a negative isotype control Ab (unfilled black hexagons), the unfractionated mixture of Db‐Fc (filled black diamonds), or the following purified fractions: Db‐Fc (filled green squares), Tb‐Fc_2_ (filled orange triangles), Hb‐Fc_3_ (filled inverted cyan triangles). Cell numbers and viability were assessed by ATP/luciferase luminescence. Points represent mean ± SEM (*n* = 3).

We produced variant 1.4 as a *V*_L_–*V*_H_ fragment (Figure S1B) fused to the N‐terminus of a human Fc domain that forms a homodimer (Adams et al., 2025). The resulting fusion protein was produced in mammalian cells and purified by Protein‐A affinity chromatography. As observed previously for variant 1.3 (Adams et al., 2025), size exclusion chromatography (SEC) revealed a primary peak corresponding to the expected dimeric Db‐Fc, along with two additional peaks corresponding to tetravalent (Tb‐Fc_2_) and hexavalent (Hb‐Fc_3_) forms (Figure 1a,b).

Following preparative SEC purification (Figure 1b), we tested the fractions in a UT‐7/Epo cell proliferation assay, a standard readout for EPOR activation (Adams et al., 2025; Erickson‐Miller et al., 2000; Komatsu et al., 1993) (Figure 1c). The unfractionated mixture showed strong agonist activity comparable to EPO. Both the Tb‐Fc_2_ and Hb‐Fc_3_ fractions were also highly active, while the Db‐Fc dimer exhibited minimal activity only at high concentrations — likely due to minor contamination by higher‐order oligomers. These results are consistent with earlier findings with variant 1.3 (Adams et al., 2025) and confirm that dimeric Dbs are inactive, whereas tetravalent Tbs and higher‐order oligomers are potent EPOR agonists.

### 2.2 Design and production of alternative tetravalent Ab formats

**FIGURE 2:**
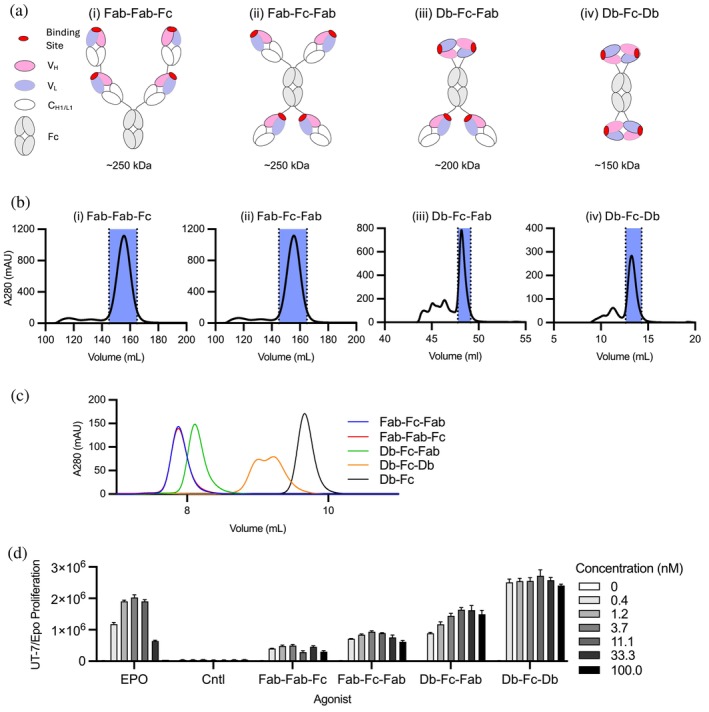
In vitro EPOR agonist activity of tetravalent Abs. (a) The following tetravalent Ab formats are shown: (i) Fab‐Fab‐Fc, (ii) Fab‐Fc‐Fab, (iii) Db‐Fc‐Fab, and (iv) Db‐Fc‐Db. The following colors are used: CDRs (red), *V*_H_ (pink), *V*_L_ (lavender), *C*_L_ and *C*_H1_ (white), and Fc (gray). (b) Preparative SEC of tetravalent Ab formats on a Superdex 200 column. Protein peaks (shaded) representing the tetravalent form were collected for (i) Fab‐Fab‐Fc, (ii) Fab‐Fc‐Fab, (iii) Db‐Fc‐Fab, and (iv) Db‐Fc‐Db. (c) Analytical SEC of tetravalent Ab fractions on a Tosoh TSK gel filtration column. Tetravalent fractions collected in (b) were analyzed for Fab‐Fab‐Fc (red), Fab‐Fc‐Fab (blue), Db‐Fc‐Fab (green), and Db‐Fc‐Db (orange). A purified bivalent Db‐Fc (black) is shown for reference. (d) UT‐7/Epo cell proliferation assays. Proliferation (*y*‐axis) was monitored in the presence of three‐fold serial dilutions (100–0.4 nM) of EPO, a negative isotype control Ab (Cntl), or the indicated purified tetravalent Ab (*x*‐axis). Bars represent mean ± SEM (*n* = 5).

Leveraging our prior experience with tetravalent Ab engineering, we developed four alternative formats to re‐engineer the heterogeneous EPOR agonist. In other contexts, each of these designs has proven amenable to purification as homogeneous, monodisperse proteins that remain stable and resistant to oligomerization and aggregation during long‐term storage. Specifically, we engineered Fab‐Fab‐Fc (Figure 2a,i), Fab‐Fc‐Fab (Figure 2a,ii), and Db‐Fc‐Fab (Figure 2a,iii) formats — previously employed in the generation of potent SARS‐CoV‐2 antagonists (Miersch et al., 2022) — as well as a Db‐Fc‐Db format (Figure 2a,iv), which we have used to develop agonists of FZD/LRP signaling (Tao et al., 2019).

Despite the structural complexity of these tetravalent Abs, all formats are constructed on a single, stable framework similar to that of Trastuzumab and several other clinically approved biologics (Sidhu et al., 2004). This shared framework allowed us to utilize standard production and purification methods commonly employed for Trastuzumab and other IgG‐class Abs. Abs were expressed via transient transfection of mammalian cells with expression plasmids encoding the appropriate heavy and light chains. Secreted Abs were then purified by Protein‐A affinity chromatography, yielding 3.7 mg/L (Db‐Fc‐Db), 17 mg/L (Db‐Fc‐Fab), 58 mg/L (Fab‐Fab‐Fc), and 90 mg/L (Fab‐Fc‐Fab).

Each Protein‐A‐purified preparation was analyzed by SEC. In each case, we observed a dominant peak at the expected retention volume for the designed format, accompanied by variable levels of faster‐eluting peaks indicative of higher‐order oligomers (Figure 2b). Preparative SEC was subsequently employed to isolate the main peak from each preparation. Reanalysis of the purified main peaks confirmed that three of the formats remained monodisperse, with no detectable higher‐order oligomers. The exception was the Db‐Fc‐Db format, which displayed a broad, bimodal peak profile (Figure 2c).

### 2.3 Assessment of EPOR agonist activity of tetravalent Abs in vitro

We evaluated the EPOR agonist activity of purified tetravalent Ab peak fractions using UT‐7/Epo cell proliferation assays (Figure 2d). Like EPO, both the Db‐Fc‐Fab and Db‐Fc‐Db formats showed high potency (EC_50_ <1 nM) and strong efficacy, with maximum luminescence signals of 2.0 × 10^6^, 1.6 × 10^6^, and 2.7 × 10^6^ RLU, respectively. Notably, neither tetravalent format showed any evidence of a hook effect (Selby, 1999), even at the highest concentrations tested. In contrast, although the Fab‐Fab‐Fc and Fab‐Fc‐Fab formats also exhibited high potency (EC_50_ <1 nM), their maximal responses were substantially lower (5.0 × 10^5^ and 9.4 × 10^5^ RLU, respectively).

These results indicate that the four tetravalent Ab formats act as potent EPOR agonists, consistent with high‐affinity, multivalent receptor engagement on the cell surface. However, efficacy varied markedly by format: the Db‐Fc‐Db exhibited the greatest activity followed by the Db‐Fc‐Fab, while the Fab‐Fab‐Fc and Fab‐Fc‐Fab formats displayed reduced efficacy. We hypothesize that the Db domains may enable more effective clustering of EPOR monomers compared to the IgGs or tetravalent Fab arrangements due to more optimal geometric configurations and proximity of the induced EPOR dimers. Collectively, these findings demonstrate that tetravalent Abs, across a range of structural formats, can activate EPOR signaling with high potency, though their efficacy is influenced by the spatial configuration and size of the antigen‐binding domains.

### 2.4 Assessment of EPOR agonist activity of tetravalent Abs in vivo

Encouraged by the potent agonist activity of the Db‐Fc‐Fab and Db‐Fc‐Db proteins observed in vitro, we next assessed their effects on erythropoiesis in vivo. To this end, we utilized knock‐in mice expressing the human *EPOR* in place of the endogenous mouse *Epor* (Divoky & Prchal, 2002; Lacy et al., 2008; Liu et al., 2007) and compared the efficacy of each tetravalent Ab with that of darbepoetin, the clinically dominant ESA (Ibbotson & Goa, 2001).

**FIGURE 3:**
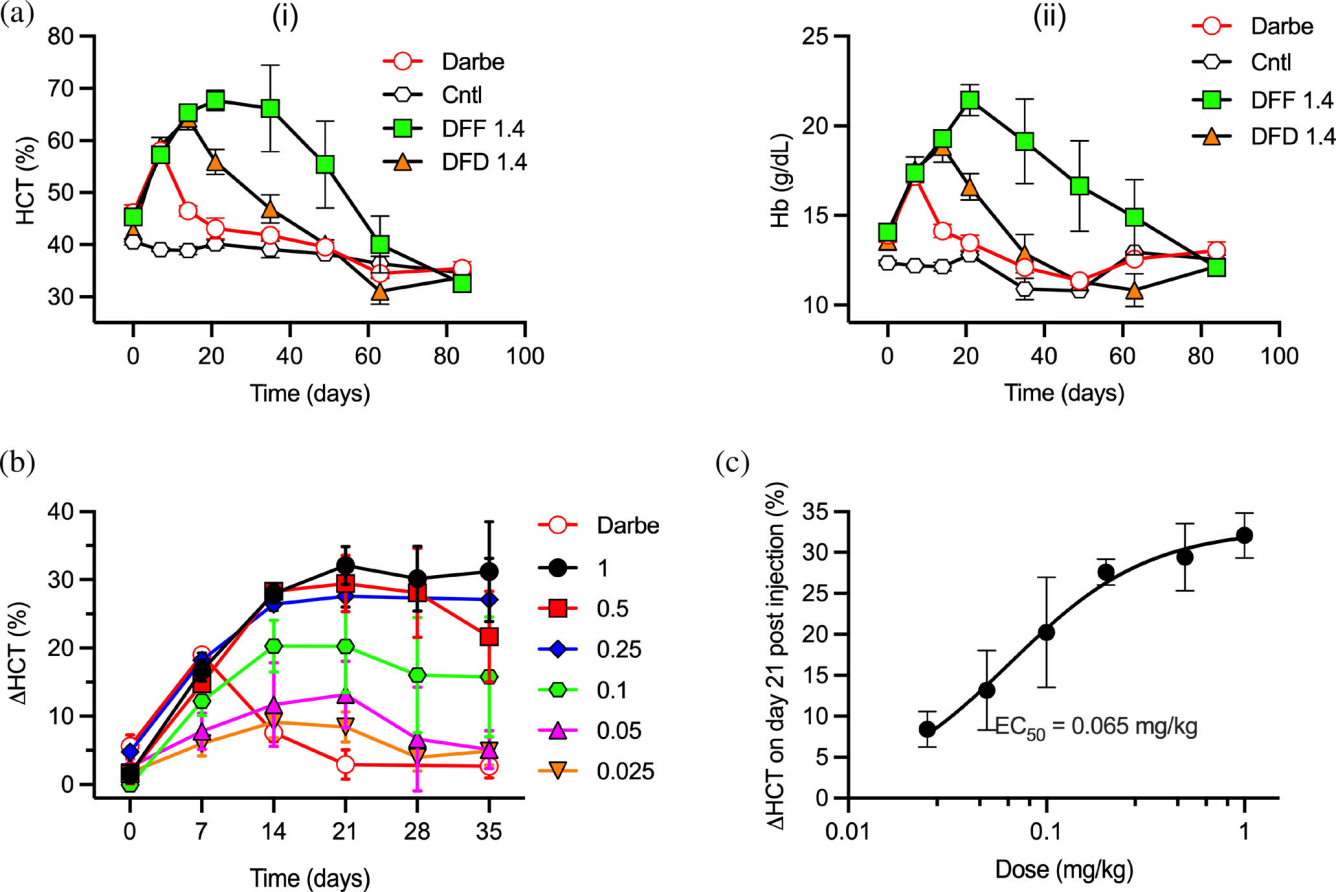
In vivo EPOR agonist activity of Db‐Fc‐Db and Db‐Fc‐Fab. (a) In vivo effects on (i) HCT and (ii) Hb in humanized EPOR mice treated by intraperitoneal injection with positive control darbepoetin (Darbe, 1 mg/kg, unfilled red circles), a negative isotype control Ab (Cntl, 0.2 mg/kg, unfilled black hexagons), Db‐Fc‐Fab 1.4 (DFF, 0.2 mg/kg, filled green squares), or Db‐Fc‐Db 1.4 (DFD, 0.2 mg/kg, filled orange triangles). Peripheral blood HCT or Hb (*y*‐axis) was assayed at various time points (*x*‐axis). Experimental points represent mean ± SEM (*n* = 3). Cntl points represent mean ± SEM (*n* = 2). (b) In vivo effects on HCT in humanized EPOR mice treated by intraperitoneal injection with darbepoetin (Darbe, 0.1 mg/kg, unfilled red circles) or indicated doses (mg/kg) of Db‐Fc‐Fab 1.4 (filled symbols). Change in peripheral blood HCT relative to day 0 (*y*‐axis) was assayed at various time points (*x*‐axis). (c) Dose response curve for change in peripheral blood HCT at day 21 relative to day 0 (*y*‐axis) following treatment with the indicated concentration of Db‐Fc‐Fab 1.4 (*x*‐axis). Data were taken from (b) and the EC_50_ value was determined by fitting to a nonlinear regression model (*R* ^2^ = 0.992) (Prism GraphPad) of the average change in HCT observed (ΔHCT) at day 21 compared to day 0. Experimental points represent mean ± SEM (*n* ≥3). Cntl points represent mean ± SEM (*n* ≥2).

A single, intraperitoneal (IP) injection of each protein increased hematocrit (HCT, Figure 3a,i) and hemoglobin (Hb, Figure 3a,ii) with onset and rate of rise comparable to darbepoetin. However, in contrast to darbepoetin, which showed peak responses by week 1 and returned to baseline within 2–3 weeks, both Abs induced significantly higher peak HCT and Hb levels, with prolonged duration of action. The prolonged response compared to darbepoetin was at least in part due to Fc‐mediated retention of the Abs in serum (Adams et al., 2025). Notably, HCT and Hb levels remained significantly elevated for over 1 month following a single dose of Db‐Fc‐Db, and for more than 2 months following Db‐Fc‐Fab administration. In both cases, HCT and Hb levels ultimately returned to baseline, confirming the reversibility of the erythropoietic response.

Encouraged by the sustained efficacy of the Db‐Fc‐Fab, we conducted a dose–response study in the same mouse model. Mice were treated with two‐fold serial dilutions of the Ab, starting at 1 mg/kg. Remarkably, even at the lowest dose tested (0.025 mg/kg), HCT levels remained significantly elevated above baseline for more than 1 month after treatment (Figure 3b). In contrast, darbepoetin induced a transient elevation in HCT at week 1, followed by a return to baseline by week 2.

The erythropoietic response to Db‐Fc‐Fab displayed clear dose‐dependency, with peak HCT values observed at week 3 and the magnitude of the response correlating closely with the dose. Plotting the change in HCT at week 3 as a function of dose revealed a linear pharmacodynamic relationship at low doses that plateaus with an in vivo EC_50_ value of 0.065 mg/kg (Figure 3c). In summary, compared to darbepoetin, the dominant ESA in clinical use, the superiority of the Db‐Fc‐Fab lies in its higher efficacy and longer duration of action.

### 2.5 Biophysical properties of Db‐Fc‐Fab and Db‐Fc‐Db tetravalent Abs

To ensure developability, biologic therapeutics must exhibit high stability and homogeneity. Drawing on analyses of Ab drugs approved by the US Food and Drug Administration (USFDA), several key developability criteria have been established to guide the selection of optimal lead candidates for clinical development (Mieczkowski et al., 2023). Accordingly, we subjected the Db‐Fc‐Fab and Db‐Fc‐Db proteins to a comprehensive panel of early‐stage developability assays.

Both proteins displayed two thermal unfolding transitions (*T*_m1_ and *T*_m2_, Table 1, Thermostability), consistent with domain‐level unfolding behavior observed in IgGs (Menzen & Friess, 2014; Vermeer & Norde, 2000). The Db‐Fc‐Fab exhibited slightly higher thermal stability than the Db‐Fc‐Db, and a higher aggregation onset temperature (*T*_agg_, Table 1, Thermostability). Dynamic light scattering (DLS) analysis revealed a larger hydrodynamic radius (Rh, Table 1) for the Db‐Fc‐Fab relative to the Db‐Fc‐Db and to the reported Rh of the IgG Bevacizumab (Akbas et al., 2018). This difference is attributable to the larger size of the Db‐Fc‐Fab, yet its Rh is within the expected range for tetravalent Abs of ~200 kDa (Lu & Zhu, 2009). Importantly, the Db‐Fc‐Fab also displayed a lower polydispersity index (PDI, Table 1), indicating a greater tendency to exist in a monodisperse state.

**TABLE 1:** Biophysical parameters for the Db‐Fc‐Fab and Db‐Fc‐Db proteins.

Protein	Thermostability (°C)			Rh (nm)	PDI	Subvisible particles (per mL)		
	*T*_m1_	*T*_m2_	*T*_agg_			2–10 μm	11–25 μm	26–100 μm
Db‐Fc‐Fab	68.5	82.1	64.4	6.8	0.089	7869	174	0
Db‐Fc‐Db	66.8	81.8	61.1	5.7	0.110	39,624	1519	238

Abbreviations: PDI, polydispersity index; Rh, hydrodynamic radius.

The most pronounced difference between the two formats was in subvisible particle (SVP) content. According to USFDA guidance, SVPs — even those under 10 μm — may contribute to immunogenicity, and while no strict upper limit is defined, minimizing SVP levels is strongly recommended during early development. The Db‐Fc‐Fab demonstrated clear superiority, with approximately five‐fold fewer SVPs in the 2–10 μm range compared to the Db‐Fc‐Db, and even more dramatic reductions in larger particles (Table 1, Subvisible Particles).

Collectively, these preliminary developability data, when considered alongside the favorable pharmacodynamic profile of the Db‐Fc‐Fab in vivo (Figure 3), support its selection over the Db‐Fc‐Db as a preferred candidate for drug development.

### 2.6 Development of stable CHO‐S cell lines for Db‐Fc‐Fab production

To enable large‐scale production of the Db‐Fc‐Fab protein, we generated stable Chinese hamster ovary (CHO‐S) cell lines using IcoCell® expression technology. DNA encoding the heavy and light chains was stably transfected into CHO‐S cells, and 13 minipools were established. Protein titers were assessed from culture supernatants (Figure S2), and the highest‐expressing minipool (MP‐14B3) was selected for further analysis.

**FIGURE 4:**
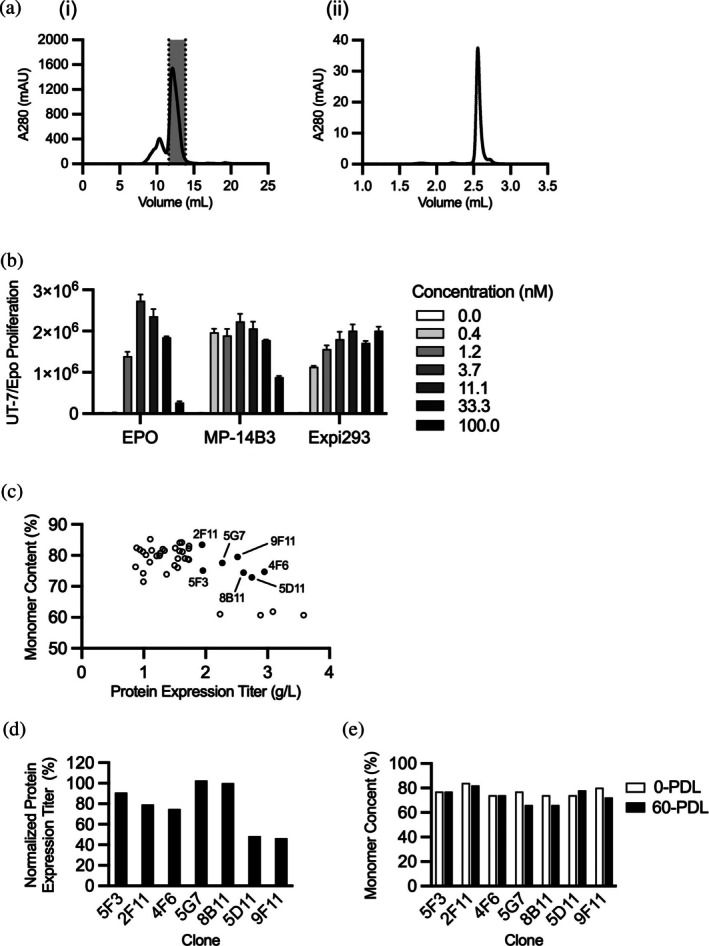
Characterization of Db‐Fc‐Fab protein produced from stable ICOCELL® cell lines. (a) Preparative SEC of Db‐Fc‐Fab protein purified by Protein‐A affinity chromatography from MP‐14B3 supernatant. (i) The protein was analyzed by SEC and the main peak fraction (shaded) was purified and (ii) reanalyzed by SEC. (b) UT‐7/Epo cell proliferation assays. Proliferation (*y*‐axis) was monitored in the presence of three‐fold serial dilutions (100–0.4 nM) of EPO or purified Db‐Fc‐Fab derived from MP‐14B3 or from transiently transfected Expi293 cells (*x*‐axis). (c) Ab expression titer (*x*‐axis) and monomer content (*y*‐axis) determined by SEC for Db‐Fc‐Fab protein obtained from 40 individual clones derived from MP‐14B3. Protein was purified by Protein‐A affinity chromatography and the monomer content was determined by analytical SEC. Labeled filled circles indicate clones that were chosen for further analysis. (d) Protein expression titer of Db‐Fc‐Fab after 60‐PDLs normalized to the titer at 0‐PDLs (*y*‐axis) for individual clones (*x*‐axis). (e) Monomer content (*y*‐axis) for Db‐Fc‐Fab purified from individual clones (*x*‐axis) at 0‐PDL (white bars) or after 60‐PDLs (black bars). Proteins were purified by Protein‐A affinity chromatography and analyzed by SEC. The monomer content is defined as the percentage of the total protein that is in the major SEC peak.

Ab secreted by MP‐14B3 was purified via Protein‐A chromatography and subjected to analytical SEC. The majority of the protein (73%) eluted as a single peak at the expected retention volume for monomeric Db‐Fc‐Fab, and earlier‐eluting material likely represented higher‐order oligomers (Figure 4a,i). The main peak was further purified by preparative SEC to >95% homogeneity and was confirmed to be stable upon reanalysis (Figure 4a,ii). Critically, the purified Db‐Fc‐Fab retained high potency and efficacy in the UT‐7/Epo proliferation assay, matching that of both recombinant EPO and transiently expressed Db‐Fc‐Fab from Expi293 cells (Figure 4b).

We isolated 40 individual clones from MP‐14B3 and evaluated each for protein yield and monomer content. Seven clones with both high yield (>1.9 g/L) and high monomer content (>73%) were selected for stability testing (Figure 4c). Clones were passaged to 60 population doublings (PDLs), and production stability was assessed by comparing yields (Figure 4d) and monomer content (Figure 4e) at 0 and 60 PDLs. Clones 5D11 and 9F11 failed to maintain sufficient yield, while 5G7 and 8B11 showed reduced monomer content. The remaining three clones (5F3, 2F11, and 4F6) maintained both high yield and high monomeric content over time and were deemed suitable for long‐term manufacturing. These results establish a robust platform for stable, large‐scale production of a clinical‐grade Db‐Fc‐Fab Ab using CHO‐S cells.

## 3 DISCUSSION

The development of potent and efficacious Abs as ESAs marks a transformative advance in the treatment of anemia, particularly in patients with low endogenous EPO such as those with CKD and related conditions. This study highlights the exceptional preclinical performance of a novel tetravalent EPOR agonist, a Db‐Fc‐Fab, which outperforms darbepoetin — the current clinical standard ESA — in efficacy and duration of action and also demonstrates manufacturing stability and suitability for clinical development.

Unlike traditional ESAs based on EPO, our tetravalent Ab avoids the limitations associated with the natural cytokine, including rapid clearance, interactions with receptors beyond EPOR, and production complexity. Building on our previous findings that demonstrated the activity of a tetravalent antibody modality (Adams et al., 2025), we describe here the engineering of a Db‐Fc‐Fab format that activates EPOR with high potency and efficacy, as evidenced by sustained in vivo responses, including elevated HCT levels that persist for over a month following a single dose. These properties suggest a potential for reduced dosing frequency and improved patient compliance — both of which are highly desirable attributes for chronic anemia management.

Importantly, while we had shown previously that a tetravalent modality was the active conformation within a mixture of species (Adams et al., 2025), we now resolve the challenges associated with production using a stable tetravalent modality. We demonstrate that the Db‐Fc‐Fab protein can be manufactured in CHO‐S cells with scalable, industry‐standard processes, and that the purified product — named EPRA‐0322 — meets key developability criteria including monodispersity and thermostability. These attributes significantly de‐risk the path to clinical translation and support the advancement of EPRA‐0322 into formal IND‐enabling studies.

Positioned as the first Ab‐based ESA to combine strong in vivo efficacy with manufacturability and pharmaceutical stability, this candidate stands out as a next‐generation biologic therapy. It builds upon our earlier foundational studies defining EPOR agonist structural requirements (Adams et al., 2025) and is complemented by ongoing in vivo pharmacology work.

As the field moves toward more innovative, targeted biologics, the tetravalent EPOR agonist described here offers significant advantages for drug development. Its best‐in‐class potency, extended duration of effect, and strong manufacturing profile make it a compelling drug candidate. We believe this platform not only addresses a large clinical need but also opens the door to a new class of Ab‐based agonists with potential applications beyond erythropoiesis.

In summary, we demonstrate that engineered tetravalent Abs — particularly the Db‐Fc‐Fab EPRA‐0322 — are potent, efficacious, and durable ESAs that outperform darbepoietin in preclinical models. The favorable biophysical properties, manufacturability, and extended in vivo activity of EPRA‐0322 establish it as a compelling next‐generation ESA with strong translational potential. These findings pave the way for advancing EPRA‐0322 into formal preclinical development and clinical evaluation for anemia associated with chronic disease.

## 4 METHODS

### 4.1 Production and purification of Ab proteins by transient transfection of Expi293 cells

Ab production and purification from transiently transfected Expi293 cells was performed as described (Adams et al., 2025). Briefly, plasmids (pSCSTa) designed to express the Ab protein were singly transfected or co‐transfected into Expi293 cells according to the manufacturer's instructions. After 5 days, cell culture medium was harvested and applied to a rProtein‐A affinity column (Cytiva GE17‐1279‐03). Ab protein was eluted with IgG elution buffer (Pierce) and immediately neutralized with 1 M Tris‐NaCl pH 8. Fractions containing eluted Ab protein were combined, concentrated, and dialyzed into PBS, pH 7.4. Ab protein was characterized for purity by SDS‐PAGE, and concentration was determined by spectrophotometry at an absorbance wavelength of 280 nm.

### 4.2 Production and purification of Ab proteins by transient transfection of CHO‐S cells

CHOEBNALT85‐1E9 cells 007 (Icosagen, Tartu, Estonia) were transfected using Reagent 007 (Icosagen, Tartu, Estonia) and cultivations were performed in CHO TF (Xell AG) medium at 37°C for the first 3 days, followed by incubation at 30°C until the end of cultivation. Supernatants were harvested at day 7 and applied to a HiTrap PrismA (Cytiva) affinity column followed by preparatory SEC using a Superdex 200 HiLoad column (Cytiva). Purified material was formulated in 10 mM L‐histidine, 0.9% sucrose, 140 mM NaCl pH 6.0.

### 4.3 Development of ICOCELL® CHO‐S based stable cell lines

To generate a stable ICOCELL® minipool, the DNA sequences encoding the heavy and light chains of the Db‐Fc‐Fab protein were codon optimized using Icosagen Cell Factory's proprietary algorithm. DNA was synthesized by Integrated DNA Technologies (gBlocks HiFi Gene Fragments) and used to construct an expression vector with the pGSN vector as the backbone. The linearized construct was electroporated into ICOCELL® cells using a Bio‐Rad Gene Pulser II apparatus supplied with a capacitance extender according to the manufacturer's instructions (Bio‐Rad Laboratories). After 2 days, the supernatants were collected, secreted Ab production was verified, and glutamine synthetase (GS)‐based metabolic selection was initiated in a 96‐well cell culture plate format in glutamine‐free selection medium. Based on cell growth after 16–19 days, the top 13 minipools were scaled up for fed‐batch production in 125‐mL shake flasks at 35‐mL scale using 4Cell XtraCHO system medium and supplement feed medium A and B, according to the manufacturer's instructions (Sartorius). The production supernatants were harvested once cell culture viability fell below 80% and the Db‐Fc‐Fab protein concentration of each minipool was determined using the Protein‐A (ProtA) high‐pressure liquid chromatography (HPLC) method.

Minipool 14B3 was single‐cell printed onto 1096‐well plates in CD CHO Medium (Thermo Fisher Scientific) by the VIPS Instrument according to the manufacturer's instructions (Advanced Instruments). ELISAs were performed to determine the expression of the Db‐Fc‐Fab protein in the supernatant and the top 40 clones were scaled up for further evaluation in a fed‐batch production. The top 7 clones with the highest monomeric titer were selected, expanded, and cryopreserved.

### 4.4 Production and purification of Ab proteins from ICOCELL® stable cells

Expression and purification from ICOCELL® minipools was performed by inoculating stable cells into the Ambr250m bioreactors (Sartorius Lab Instruments) at a density of 7.5 × 10^5^ cells/mL in 180 mL of Sartorius 4Cell XtraCHO production medium. Starting at day 2, the production cultures were supplemented once a day with 3% Sartorius feed medium A and 0.3% Sartorius feed medium B of the initial culture volume. The agitation speed was set to 395 rpm, the pH was set at 7.2, deadband ±0.2, and the dissolved oxygen setpoint was 40%. To prevent foam formation in the bioreactors, 3% Antifoam C Emulsion solution (Sigma‐Aldrich) was used as required. The cultures were harvested when viability fell below 85%. The supernatant was centrifuged, filtered through a 0.45‐μm PES filter, and loaded onto a MabSelect PrismA Protein‐A column. Elution was performed with 0.1 M sodium citrate, pH 3.3 and immediately neutralized with 1.5 M Tris–HCl, pH 8.8. Fractions containing eluted protein were combined and dialyzed into PBS, pH 7.4.

For individual clones, fed‐batch culturing was conducted in 125‐mL shake flasks with a starting working volume of 25 mL HyClone ActiPro production medium. On day 0, cultures were inoculated at 7.5 × 10^5^ viable cells/mL. Starting at day 2, the production cultures were supplemented once a day with 3% Cell Boost 7A and 0.3% Cell Boost 7B of initial culture volume. Cultures were maintained at 37°C in an incubator with 8% CO_2_ and shaking at 120 rpm, followed by a temperature shift to 32°C and shaking at 160 rpm on day 4. The cultures were harvested when the cell viability dropped below 80% and were subjected to Protein‐A affinity purification using the KingFisher Flex system and the Mag Sepharose PrismA magnetic bead resin (Cytiva, 1755000) according to the manufacturer's instructions.

### 4.5 Sandwich ELISA and HPLC quantification of Db‐Fc‐Fab protein

CHO mini‐pools were initially screened for relative expression of Db‐Fc‐Fab. To quantify the anti‐EPOR Db‐Fc‐Fab concentration in cell culture supernatant, a direct sandwich ELISA method was implemented. His‐tagged EPOR (Sino Biological, Inc.) at 1 μg/mL in Dulbecco's PBS buffer was immobilized on 96‐well Maxisorp plates for 24 h at 4°C. The plates were blocked with PBS, 0.5% BSA for 1 h at room temperature and washed five times with PBS. Cell culture supernatant samples and a serial dilution of a standard preparation of purified Db‐Fc‐Fab standard were added and incubated with the immobilized EPOR. An enzyme‐conjugated detection Ab (Peroxidase AffiniPure Donkey Anti‐Human IgG (H + L), Jackson ImmunoResearch) was applied in a 1:10,000 dilution for detection using TMB Solution VII substrate (Biopanda Diagnostics) and measured spectrophotometrically at 450 nm. GraphPad Software was used to calculate the Db‐Fc‐Fab Ab concentrations in the samples using standard curve extrapolation.

To calculate the mini‐pool titer, ProtA HPLC was performed on an Agilent Infinity II HPLC system using the following conditions: Agilent Bio‐Monolith rProtein‐A p/n 5190‐6903, 50 mM Na‐Phosphate pH 7.5 binding buffer eluent A, 5% acetic acid, pH 2.5 eluting buffer eluent B, 280 nm UV detection, 1.5 mL/min flow rate, and 5 μL injection volume. The gradient profile was: 0–0.4 min, 0% B (binding); 0.5–1.7 min, 100% B (elution); 1.8–3.0 min, 0% B (reconditioning). A calibration curve was constructed by injecting a Db‐Fc‐Fab protein standard in quantities of 0.625–20 μg. Peak area versus injection quantity was plotted. Cell culture supernatant samples were diluted 4‐fold in binding buffer and 5 μL injections were used. The calibration curve was used to interpolate cell supernatant sample concentrations based on measured peak areas.

### 4.6 Analytical SEC

Analytical SEC experiments were performed on an Agilent Infinity II HPLC system, using a TSKgel UP‐SW3000 250A, 4.6 × 300 mm, 2‐μm column (Tosoh, p/n 0023448) with a Guard Column (Tosoh, p/n 0023450). 2–4 μg of Protein‐A‐captured samples were loaded on the column and separation was performed using 0.2 M potassium phosphate, 0.25 M KCl pH 6.8, acetonitrile 5% mobile phase with a flow rate of 0.35 mL/min at 25°C. From analytical HPLC‐SEC chromatograms, percentile share of individual peak populations was compared to the total area of integrated peaks and expressed as Area %.

### 4.7 Preparative SEC

For material collected from transiently transfected Expi293 cell cultures, preparative SEC was performed as previously described (Adams et al., 2025). Material from MP‐14B3 minipool purified by Protein‐A (~100 mg) was concentrated to a volume of 10 mL and was run at a flow rate of 2.6 mL/min on a HiLoad 26/600 Superdex 200 column (Cytiva, 28989336) that was equilibrated with de‐gassed 10 mM histidine, 0.9% sucrose, 140 mM NaCl pH 6.0 buffer. Fractions were collected manually based on the chromatographic profile.

### 4.8 Differential scanning fluorimetry and dynamic light scattering

Differential scanning fluorimetry (DSF) and DLS experiments were performed by HTD Biosystems (USA) with material provided by EPOK Therapeutics, Inc. Protein samples were diluted to 1 mg/mL in HNS buffer (140 mM NaCl, 0.9% sucrose, 10 mM histidine pH 6.0). Approximately 10 mL of sample was loaded onto the Prometheus Panta instrument. For DSF measurements, samples were run at a ramp rate of 1.5°C/min from 20 to 95°C and fluorescence signals at 330 and 350 nm were collected for 500 ms per capillary in each round. *T*_agg_ and *T*_m_ were determined by calculating the ratio of tryptophan emission at 350 and 330 nm and taking the second derivative of the ratio during the thermal shifts as well as the initial condition. For DLS measurements, 10 acquisitions of 5,000 ms were collected, with each acquisition being evaluated separately and averaged if it met the quality parameters. All measurements were made at 405 nm and each DLS acquisition was automatically analyzed for Cumulant fit that models the autocorrelation function (ACF) using an average diffusion coefficient to obtain a single averaged hydrodynamic radius (RH) with a single PDI.

### 4.9 Sub‐visible particle analysis

Dynamic imaging particle analysis experiments were performed by HTD Biosystems (USA) with material provided by EPOK Therapeutics, Inc. Protein samples were diluted to 1 mg/mL in HNS buffer (140 mM NaCl, 0.9% sucrose, 10 mM histidine pH 6.0). The FlowCam 8000 series was used to perform microscopic particle measurements. A 10× objective was used with the FOV80 (80 μm depth × 700 μm width). 150 μL of sample was placed in the sample inlet port for autorun at a flow rate of 150 μL/min. The camera recorded digital images of the particles, and the shape and size of each particle were measured as it moved down the flow cell. The data were analyzed by removing any artifacts, bubbles, duplicate images. The images were analyzed with the FlowCam imaging software and particles were segregated into three bins based on size, as follows: 2–10 μm, 10–25 μm, and 25–100 μm. For each bin, the results were presented as the number of particles per mL.

### 4.10 Stability studies

Frozen clones were revived from a vial of cryopreserved stock by incubating each frozen vial in a CO_2_ incubator at 37°C until thawed. The cell culture was immediately diluted into 20 mL of pre‐warmed HyClone ActiPro production medium. The cryoprotectant was removed by centrifuging the cells at 200 × *g* for 5 min at ambient temperature. The supernatant was discarded, and the cells were resuspended in 25 mL of fresh, pre‐warmed HyClone ActiPro production medium.

Each thawed culture was passaged regularly 2–3 times per week. During the subculturing process, the approximate number of doublings that the cell population had undergone since the previous subculturing was counted, and it was expressed as a PDL.

When the cell clones reached 20‐PDL, the cultures were subjected to a fed‐batch production to assess expression. Passaging continued with the remaining cell culture until 60‐PDL, at which point another fed‐batch production was initiated. At 0‐PDL, the cell clones were revived from a vial of the cryopreserved cell stock and subcultured for two passages before the fed‐batch production process. Each fed‐batch production was conducted at a small scale (25 mL) in 125‐mL shake flasks. Ab concentrations in harvested production supernatants at different timepoints were determined using the ProtA HPLC method. The monomeric Db‐Fc‐Fab content in the Protein‐A affinity chromatography material was determined by analytical SEC.

### 4.11 UT7/Epo cell proliferation assay

UT‐7/Epo cells were cultured in DMEM (ThermoFisher, Cat. No. 11995‐073) supplemented with 10% fetal bovine serum (FBS) and 2 U/mL EPO (Cell Sciences, Cat. No. CRE600D). Cultures were passaged at a 1:5 ratio every few days to maintain exponential growth. For the proliferation assay, cells were prepared 1 day before ligand exposure. UT‐7/Epo cells were harvested by centrifugation at 1200 rpm for 5 min, washed twice with PBS, and resuspended in serum‐free DMEM (starvation medium). Cells were then seeded at 10,000 cells per well in 90 μL of serum‐free medium in 96‐well plates and incubated overnight at 37°C with 5% CO_2_. The following day, cells were treated with either recombinant EPO or the indicated Abs at the desired concentrations. Ligands were prepared as 10× stocks in serum‐free medium, and 10 μL of the diluted EPO or Ab solution was added to each well (100 μL). Cells were cultured for 3 days at 37°C with 5% CO_2_. Proliferation was quantified using the Promega CellTiter assay (Cat. No. G9242) according to the manufacturer's instructions.

### 4.12 Animal studies

All animal procedures were approved by the Institutional Animal Care and Use Committee at the University of Texas Southwestern Medical Center in Dallas, TX, and conducted in accordance with institutional and national guidelines. All experiments with humanized EPOR knock‐in mice were performed as described elsewhere with modifications (Adams et al., 2025). Briefly, for single‐concentration, time‐course experiments, on day 0, mice were injected intraperitoneally (IP) with 1 mg/kg darbepoetin or 0.2 mg/kg of an isotype control Ab, Db‐Fc‐Fab 1.4, or Db‐Fc‐Db 1.4. Peripheral blood HCT and Hb were assayed on days 0 (before injection), 7, 14, 21, 35, 49, 63, and 84. For the dose–response study, 1 mg/kg darbepoietin or various doses of Db‐Fc‐Fab were administered via IP injection. HCT was measured every 7 days up to day 35. All blood counts were obtained using a Hemavet 950 (Drew Scientific). A dose–response curve was calculated by plotting the percent increase in HCT at day 21 relative to day 0 of each cohort and then fitted to a nonlinear regression model using Graphpad Prism software (Boston, MA, USA).

## AUTHOR CONTRIBUTIONS

**Jarrett J. Adams:** Conceptualization; writing – original draft; data curation; formal analysis; methodology; supervision. **Levi L. Blazer:** Conceptualization; methodology; formal analysis; data curation; supervision; investigation; writing – original draft. **Jacky Chung:** Writing – original draft; investigation; formal analysis; supervision; data curation; methodology; conceptualization; project administration. **Minoo Karimi:** Methodology; validation; data curation; formal analysis. **Taylor Davidson:** Investigation; methodology; formal analysis; data curation; supervision; writing – original draft. **Bailey Blair:** Investigation; methodology; formal analysis. **Carlos Waddle:** Investigation; methodology; formal analysis. **Craig A. Hokanson:** Methodology; investigation. **Heather A. Bruce:** Investigation; methodology. **Alexander U. Singer:** Investigation; methodology. **Eva‐Maria Tombak:** Investigation; methodology; formal analysis. **Kiira Gildemann:** Investigation; methodology; formal analysis. **Nele Tamberg:** Investigation; methodology; formal analysis. **Kaja Kiiver:** Investigation; methodology; formal analysis. **Mart Ustav Jr:** Supervision; project administration. **Yue Ma:** Investigation; methodology; formal analysis. **Luigi Colombo:** Formal analysis; data curation; project administration. **Lily Jun‐Shen Huang:** Investigation; methodology; formal analysis. **Stephen W. Michnick:** Supervision; formal analysis. **Orson W. Moe:** Conceptualization; investigation; formal analysis; supervision; project administration; data curation; writing – original draft. **Sachdev S. Sidhu:** Conceptualization; investigation; writing – original draft; methodology; formal analysis; supervision; project administration; data curation.

## Supporting information


**Data S1:** Figures.

**Data S2:** Supporting Information.

## Data Availability

The data that support the findings of this study are available from the corresponding author upon reasonable request.
