# Supplementary material for: Tetravalent antibodies are more potent and efficacious erythropoiesis‐stimulating agents than erythropoietin in vivo
Source: Protein Sci. 2026 Jan 20;35(2):e70462. doi: 10.1002/pro.70462 (PMC12817467; doi:10.1002/pro.70462)
Supplement: Supplementary file 1 — Data S1: Figures. [file PRO-35-e70462-s001.pdf]

(A)

Ab	CDR-L3 107	108	109	114	115	116	CDR-H1 30	35	36	37	38	39	CDR-H2 55	56	57	58	59	62	63	64	65	66	CDR-H3 107	108	109	114	115	116
1	S	S	Y	S	L	I	L	Y	S	Y	Y	I	S	I	S	P	Y	Y	S	Y	T	Y	H	G	Y	G	A	M
2	A		Y	W	P	I	L	S	S	Y	Y	I	S	I	S	P	Y	Y	S	Y	T	Y	H	S	Y	A	A	L
1.1	S	S	Y	S	L	I	L	R	S	Y	Y	M	S	I	S	P	Y	Y	S	Y	T	Y	H	G	Y	G	A	M
1.2	S	S	N	F	L	I	L	R	S	Y	Y	M	S	I	A	P	Y	H	G	Y	T	Y	H	G	Y	G	A	L
1.4	S	S	Q	F	L	I	L	D	S	Y	Y	M	S	I	A	P	Y	H	G	Y	T	Y	H	G	Y	G	A	L

(B)

DIQMTQSPSSLSASVGDRVTITCRASQ**SVSSA**VAWYQQKPGKAPKLLIY**SASSLYS**GVPSRFSGSRSGTD
FTLTISSLQPEDFATYYCQQ**SSQFLI**TFGQGTKVEIKGGGGGEVQLVESGGGLVQPGGSLRLSCAASGFN
LDSYYMHWVRQAPGKGLEWVA**SIAPYHGYTY**YADSVKGRFTISADTSKNTAYLQMNSLRAEDTAVYYCAR
HGYGALDYWGQIGDVSGLANGNGATGGTLVTVSS

Supplementary Figure S1

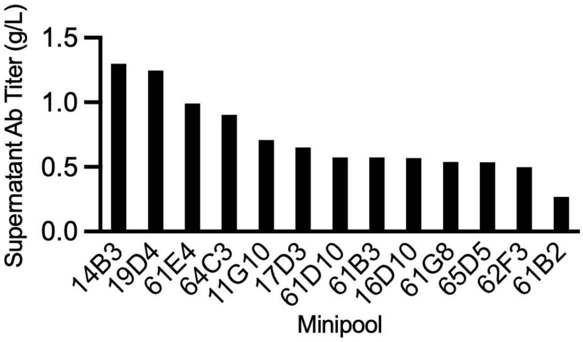

Supplementary Figure S2
